# Minimal Tissue Reaction after Chronic Subdural Electrode Implantation for Fully Implantable Brain–Machine Interfaces

**DOI:** 10.3390/s21010178

**Published:** 2020-12-29

**Authors:** Tianfang Yan, Seiji Kameda, Katsuyoshi Suzuki, Taro Kaiju, Masato Inoue, Takafumi Suzuki, Masayuki Hirata

**Affiliations:** 1 Department of Neurological Diagnosis and Restoration, Osaka University Graduate School of Medicine, Suita 565-0871, Japan; tianfang_yan@ndr.med.osaka-u.ac.jp (T.Y.); s-kameda@ndr.med.osaka-u.ac.jp (S.K.); 2 Global Center for Medical Engineering and Informatics, Osaka University, Suita 565-0871, Japan; 3 Department of Neurosurgery, Osaka University Graduate School of Medicine, Suita 565-0871, Japan; 4 Ogino Memorial Laboratory, Nihon Kohden Corporation, Tokorozawa 359-0037, Japan; Katsuyoshi_Suzuki@mb4.nkc.co.jp; 5 Center for Information and Neural Networks, National Institute of Information and Communications Technology, Suita 565-0871, Japan; kaiju@nict.go.jp (T.K.); minoue@nict.go.jp (M.I.); t.suzuki@nict.go.jp (T.S.)

**Keywords:** chronic tissue reaction, subdural electrode, implantable device, brain–machine interface, animal model

## Abstract

There is a growing interest in the use of electrocorticographic (ECoG) signals in brain–machine interfaces (BMIs). However, there is still a lack of studies involving the long-term evaluation of the tissue response related to electrode implantation. Here, we investigated biocompatibility, including chronic tissue response to subdural electrodes and a fully implantable wireless BMI device. We implanted a half-sized fully implantable device with subdural electrodes in six beagles for 6 months. Histological analysis of the surrounding tissues, including the dural membrane and cortices, was performed to evaluate the effects of chronic implantation. Our results showed no adverse events, including infectious signs, throughout the 6-month implantation period. Thick connective tissue proliferation was found in the surrounding tissues in the epidural space and subcutaneous space. Quantitative measures of subdural reactive tissues showed minimal encapsulation between the electrodes and the underlying cortex. Immunohistochemical evaluation showed no significant difference in the cell densities of neurons, astrocytes, and microglia between the implanted sites and contralateral sites. In conclusion, we established a beagle model to evaluate cortical implantable devices. We confirmed that a fully implantable wireless device and subdural electrodes could be stably maintained with sufficient biocompatibility in vivo.

## 1. Introduction

The brain–machine interface (BMI) is a rapidly developing technology that utilizes brain signals to control external devices for communication with others and machine interactions. Thus, BMIs have the potential to improve the quality of life in patients with severe motor impairments, such as those with amyotrophic lateral sclerosis (ALS), brainstem stroke, spinal cord injury, muscular dystrophy, Parkinson’s disease, and cerebral palsy [1].

BMIs encompass several types of recording modalities according to their invasiveness. Thus far, electroencephalography (EEG), electrocorticography (ECoG), and single-unit spiking activity (SUA) are the most popular methods used in BMI research aiming at clinical use. Compared to traditional scalp EEG electrodes, ECoG electrodes are placed either epidurally or subdurally to record cortical activities and, hence, have a distinct advantage in robustness to external noise, increased signal-to-noise ratio, sensitivity to high frequency band activity, and spatial resolution [2]. SUA recorded from intra-cortical micro-needle electrodes offers the highest spatiotemporal resolution and greatly contributed to progress in BMI research [3,4]. However, the implantation of micro-needles disrupts the blood–brain barrier (BBB) and induces both acute and chronic inflammatory tissue reactions, gradually leading to neuronal degeneration and fewer neurons in the vicinity of electrodes, and thereby, reduced signal quality [5,6,7]. This has considerable risk that hinders the long-term stable use of BMI systems in clinical settings. In terms of the long term stability of the signal quality recorded from electrodes for clinical application of implantable BMI devices, we focused on the subdural placement of ECoG electrodes as possibly the best method as it has better signal quality than EEG and better long-term stability of signal quality than intracortical electrode arrays [8].

ECoG recording has been commonly used in clinical evaluations as an invasive but the most reliable and final diagnostic method to identify epileptic foci and/or brain functions for surgical treatment of intractable epilepsy. Moreover, there is a growing interest in the use of chronic subdural electrodes in BMIs and neuroprosthetics. Studies of human and non-human primates have demonstrated that the ECoG signal is a potential source to encode neural information on two- and three-dimensional hand movements and precise language synthesis [9,10,11,12,13]. Although the promising application of ECoG in BMIs and neuroprosthetics has garnered attention, there is still a lack of research on the long-term evaluation of chronic host-tissue response associated with subdural ECoG systems [14]. Clinically, subdural electrode grids are generally implanted for at most 30 days in patients for epilepsy monitoring, thus limiting the timescale of the histological analysis of subsequent surgical resection. A few longer-term investigations were reported in animal models. These studies reported the proliferation of fibrosis and thickening of the dural membrane around implantation sites and minimal changes in the cortical tissue, 1 or several months after implantation [15,16]. Although the results of fibrosis and dural thickening were considered to lead to decreased signal quality [17,18], it still needs further investigation. In addition, most of these previous studies did not use fully implantable wireless systems but wired recording systems, which might increase the inflammatory reaction due to minor or major infection via leads penetrating the skin, particularly for implantations of extended durations. These wired devices also limit patient activities and hinder the expansion of their practical application. Therefore, we developed a fully implantable wireless ECoG device based on a previously reported system, W-HERBS (Wireless Human ECoG-based Real-time BMI System) [8]. We aim to apply the device in long-term medical implantation, real-time recording, and wireless transmission of the ECoG signals to external machine interfaces. Therefore, in the current study, we established a dog model to explore the biocompatibility and chronic effect on tissues around the implanted electrodes and recording system over a 6-month period. Dogs are generally easy to manage and are submissive to people, which allowed us to perform further neurological experiments [19,20]. Our results showed that neuronal density was unaffected after subdural implantation of electrodes for 6 months. Morphological changes in microglia and astrocytes reflecting an inflammatory response were not observed under the implant.

## 2. Materials and Methods

This study was approved by the Osaka University Animal Experimentation Committee and was in accordance with the Animal Research Guidelines of Osaka University. 

### 2.1. Implanted Device

**Figure 1:**
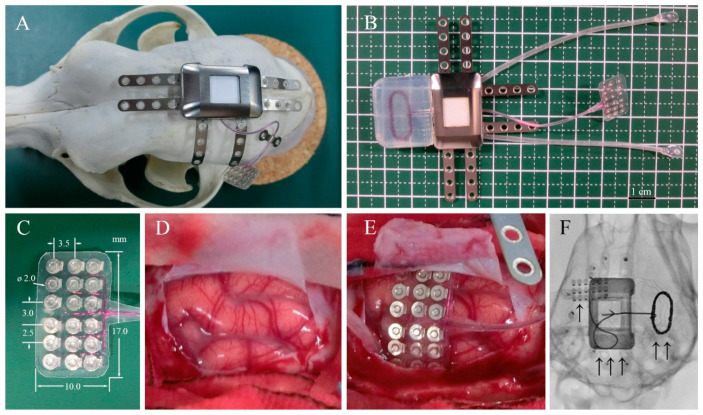
Device system and implantation. (**A**) Surgical placement of the half-sized ECoG device on the dog skull. (**B**) ECoG recording system, including a wireless charging coil, titanium case, connecting cables, and subdural electrode arrays. (**C**) Eighteen-channel subdural electrode array encapsulated in silicone rubber. (**D**) Left somatosensory cortex of the dog. (**E**) Placement of the array over the cortical surface. (**F**) Localization of the implanted ECoG recording system: “↑” platinum electrode array over the left somatosensory cortex, “↑ ↑” wireless charging coil, and “↑ ↑ ↑” titanium case in subcutaneous space.

Adult beagle dogs were used in this study. The skull size of adult beagle dogs is approximately half of that of humans. Therefore, we used a half-sized model for implantation using the same materials and manufacturing methods as those used for human implants. The design and appearance of the half-sized model of the fully implantable wireless ECoG recording device are shown in Figure 1B. The half-sized model contained a titanium casing, charging coil unit, and ECoG grid electrodes with reference and grand electrodes. The casing, 27 × 18 × 8 mm in size, was designed to be implanted unilaterally between the skull and the temporal muscle and had six titanium plates for fixation to the skull using titanium screws (Figure 1A). The charging coil unit was tightly attached to the casing. The coil unit encapsulated in silicone rubber was designed to be implanted under the dissected fascial membrane of the contralateral temporal muscle. The ECoG grid electrodes with 18-channel platinum electrodes were designed to be placed subdurally, directly on the cerebral cortical surface with the arachnoid membrane. The platinum electrodes, 2 mm in diameter, were located in a 3 × 6 grid configuration between the two silicone sheets with the interelectrode spacing of 2.5–3.5 mm according to a previous study that found that ECoG signals are recorded from cortical activity in the area 2–3 mm from the electrodes [21].

### 2.2. Implantation

All surgical procedures for subdural electrode and device implantation were performed by one neurosurgeon (MH). Six healthy male beagle dogs (three from TOYO, 12–15 months old; three from NALC, 20–21 months old) weighing 11–13 kg were used. The half-sized electrodes were surgically implanted. The grid electrodes were placed subdurally over the left hemisphere, while the contralateral hemisphere served as the control.

After inducing general anesthesia using propofol (10 mL; 1 mL/kg, iv., Pfizer, Groton, MA, USA), each dog was intubated, and the respiration was controlled using mechanical ventilation. Isoflurane (1–5%, Mylan, Canonsburg, PA, USA) was also used during the surgical procedures. Each dog was placed prone on the operating table, and the skull was stabilized in a fixation frame with four head pins fixed just under the bilateral zygomatic arch roots and maxillary bones. Standard aseptic conditions were guaranteed during the surgical procedures. A J-shaped skin incision was made from 3 cm posterior to the nasion to the inion in the midline then curved laterally, extending to 1 cm medial to the left ear lobe. Bleeding was carefully coagulated using a bipolar coagulator. The left temporal muscle was stripped and folded to sufficiently expose the left frontotemporoparietal skull surface. The subfascial space of the right temporal muscle was dissected widely to the right zygomatic bone to slip the charging coil under the subfascial space. Then, a 30 × 20-mm square craniotomy was performed using a surgical drill over the left frontoparietal bone under the microscope (OPMI6-SFCXY, ZEISS). The left dural membrane was cut and opened in a horseshoe shape, and the dural flap was folded to expose an approximately 8 × 15-mm area of cortical surface. After identification of the somatosensory cortex, the 18-channel grid electrodes (8.0 × 15.0 mm) were gently placed subdurally over the left somatosensory cortex. After electrode implantation, the dural membrane was sutured water-tightly using a 6-0 thread. An artificial dural membrane (dura wave, Gunze, Japan) was placed, and dural sealant (Adherus, Stryker, Kalamazoo, MI, USA) was used to ensure the water-tightness of the dural closure. The bone flap was placed and fixed with two pairs of titanium plates and screws. The half-sized titanium casing (Figure 1, Nihon Kohden Co., Tokyo, Japan) was affixed on the left parietal by two screws on each of the four titanium fixation plates, meanwhile the accessory charging coil covered by silicone was slipped into the subfascial space made beforehand. The dead space between the casing and the skull was filled with resin (Unifast, GC Corporation, Tokyo, Japan). Finally, the surgical space was carefully flushed to reduce the risk of infection. The left temporal muscle, galea aponeurotia, and skin were sutured to fully cover the device using a 3-0 absorbable thread. Postoperative X-rays were performed to confirm the location of the electrodes and device.

### 2.3. Behavioral Observation after Implantation

We observed behaviors including motor weakness and food intakes every day. In addition, we monitored behaviors of beagle dogs using an infrared camera 24 h a day and 7 days a week.

### 2.4. Removal

Six months after the implantation, all dogs were euthanized and their whole bodies were perfused transcardially with 10% formalin. After formalin fixation, we carefully removed the implanted device using a microscope. After drilling out the filled resin, the titanium casing and charging coil were removed. Subsequently, the bone flap was removed, the dural membrane was carefully exposed, and the sutures were cut open. In order to avoid damaging the brain tissue, we extracted the grid electrodes using a surgical microscope. The whole brain with the dural membrane and the tissues surrounding the implanted casing and coil unit were then fixed in 10% formalin and embedded in paraffin for further histological evaluation.

### 2.5. Histological Analysis

The cortical and surrounding tissues from both implanted (left) and non-implanted (control, right) sides were sectioned coronally for comparison. All slides were processed using the same procedures to minimize operation errors. Sections were collected and processed for hematoxylin-eosin (HE) staining and immunohistochemical staining for neuronal nuclei (NeuN), glial fibrillary acidic protein (GFAP), and ionized calcium-binding adapt molecule 1 (Iba-1). Images of HE-stained tissue sections were captured using a fluorescence microscope (BZ-X700, Keyence, Osaka, Japan) at 20× magnification, manually outlined, and quantitatively measured using BZ-X700 software. The encapsulation tissues from the ventral and dorsal sites, as well as the control dural membrane, were identified under the microscope; the tissue thicknesses were then determined by averaging 10 sampling points on each section (in total, *n* = 60 per group). The comparisons between ventral encapsulation, dorsal encapsulation, and control groups were performed using the paired *t*-test.

### 2.6. Statistical Analyses

For all statistical analyses, Prism v8.0 (GraphPad Software Inc., La Jolla, CA, USA) was used. The data are shown as mean ± SD, and the level of statistical significance was set at *p* < 0.05. Differences in cell densities between each group and the control group were assessed using the paired-samples *t*-test.

## 3. Results

**Figure 2:**
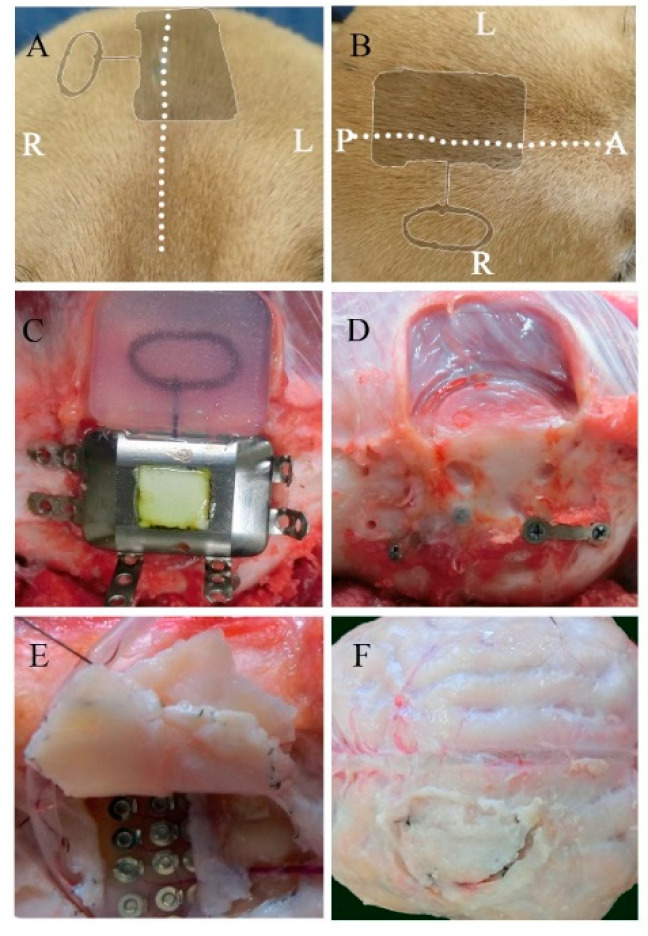
Macroscopic view of the implanted device and the surrounding tissues 6 months after implantation. Postoperative appearance of the scalp from a coronal (**A**) and transverse view (**B**) (the dotted line represents the incision line; the shadow represents the position of the implanted device). The titanium case and charging coil 6 months after implantation (**C**). Surrounding tissue and skull surface in contact with the device (**D**). The dural membrane in contact with the array after 6 months (**E**,**F**).

During the 6-month implantation period, no adverse effects were observed. No abnormal symptomatic motor behaviors were observed after implantation. No infectious signs were observed. Figure 2A,B shows the external appearance of the scalp with the implanted device 6 months after implantation. The wounds healed well, and no postoperative complications occurred. As shown in Figure 2C,D, visual inspection during device removal showed that the titanium casing and coil unit were encapsulated by connective tissues, which was a normal response to postoperative scar tissue healing. There were no apparent macroscopic signs of infection, abnormal inflammatory reaction, or necrosis related to prolonged contact with the implantable device. After removal of the skull bone flap, severe thick connective tissue formation was identified in the dural membrane (Figure 2E,F). The dural membrane was completely closed. We gently opened the dural membrane by cutting the sutures. The grid electrodes were encapsulated by connective tissue. The thickness of the subdural capsule surrounding the electrodes on the side facing the dural membrane was less than that of the epidural proliferated connective tissue on the dural membrane. The thickness of the subdural capsule was minimal or not observed on the side facing the cortical surface. The proliferated fibrous tissue tightly adhered to the dural membrane. The grid electrodes pressed against the parenchyma, causing local mechanical depression of the brain under the electrodes. Microscopic inspection of the grid electrodes did not reveal adhesion to the underlying cortex. Tiny spotty yellow pigmentations were observed in a dog, reflecting hemosiderin deposition on the surface of the electrodes, which was probably related to microbleeds during implantation in the subdural space. In order to further investigate the long-term foreign body responses and effects of chronic implantation, we performed a series of histological analyses of the surrounding tissue, dural membrane, leptomeninges, and cortex.

**Figure 3:**
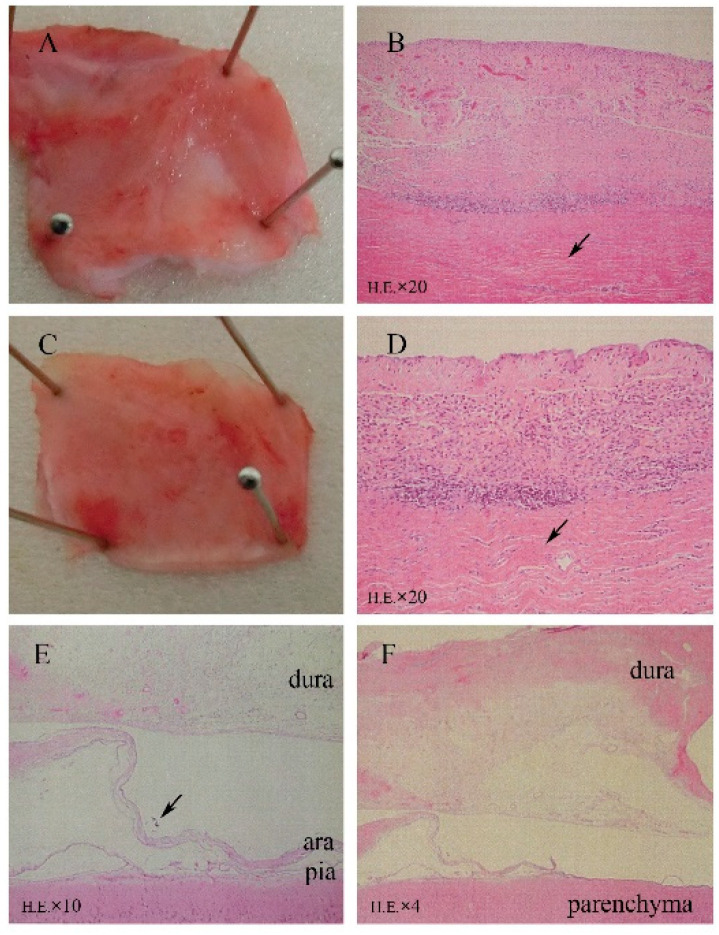
Microscopic analysis (HE staining) revealing minimal tissue reactions to the 6-month implantation of a subdural ECoG recording device. (**A**,**B**) Surrounding tissue covering the titanium case. (**C**,**D**) Tissue covering the silicone coil rubber. HE staining revealed fibrosis, mild angiogenesis, and inflammatory cell infiltration. (**E**) Implantation area with mechanically depressed arachnoid (↑) and normal leptomeningeal structures (HE). (**F**) Fibrosis, angiogenesis, and mild inflammatory cell infiltration in array-covered dura matter.

As shown in Figure 3A–D, HE staining of sections revealed fibrosis, mild inflammatory cell infiltration, and mild angiogenesis in the subcutaneous tissues surrounding the titanium case and silicone-covered coil unit. However, these inflammatory reactions were considered normal foreign body responses to chronic implantation, and the device was demonstrated to be biocompatible in the subcutaneous environment. Thick fibrous proliferation was observed epidurally on the dural membrane. The effects of electrode placement under the dural membrane facing the electrodes are shown in Figure 3E,F, with mild angiogenesis and fibrosis in all dogs, inflammatory cell infiltration in five dogs, microbleeds in one dog, and mild edema of the dural membranes in three dogs. In the epidural space, the superficial layer of the dural membrane contained more dense fibrous tissue, which was the result of fibrous proliferation in response to tissue damage. While in the subdural space, tissue sections showed that arachnoid membrane was locally depressed by the occupying effect of grid electrodes. However, the leptomeninges maintained a normal shape and structural integrity, which demonstrated that the BBB was not affected by subdural implantation (Figure 3E). There were no abnormal neurons or glial cells observed in the cortex under the grid electrodes.

**Figure 4:**
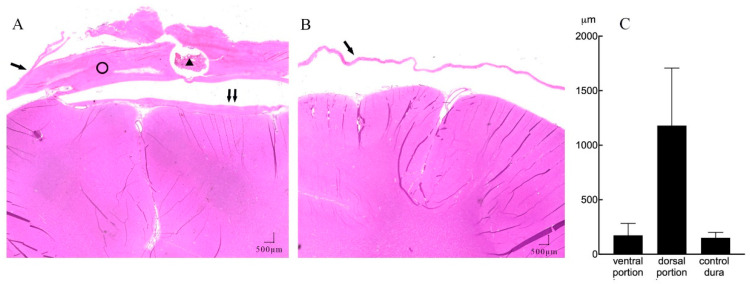
A representative example of subdural reactions surrounding the ECoG array (HE staining, 20 × magnification). (**A**) Coronal section of the implant site showing mechanical compression of the cortical tissue, with minimal reactive tissue under the electrode (↑↑) and thick fibrosis between the implanted electrode array and dura membrane (ring). There is a section of the lumen of the leads (triangle) lying inside the dorsal portion of the tissue. (**B**) Contralateral hemisphere with normal dura membrane (↑) and normal brain tissue. (**C**) Mean thickness of the dorsal, ventral encapsulation, and control dural membrane.

Further quantitative measures of tissue thickness were performed as shown in Figure 4A,B. On microscopy, we found the implanted subdural ECoG arrays were encapsulated by chronic reactive tissue. The dorsal encapsulation (between the dural membrane and array) gradually became thicker from the edge to the center part, while its thickness was much more than that of the ventral encapsulation (between the array and arachnoid mater). We then averaged the thicknesses of fibrous tissue from the pictures and compared it with that of the contralateral dural membrane. As demonstrated in Figure 4C, the dorsal encapsulation (1179 ± 527 μm) was significantly thicker than the ventral encapsulation (173 ± 109 μm) (*t*-test, *p* < 0.001) and the contralateral dural membrane (150 ± 49 μm) (*t*-test, *p* < 0.001). 

**Figure 5:**
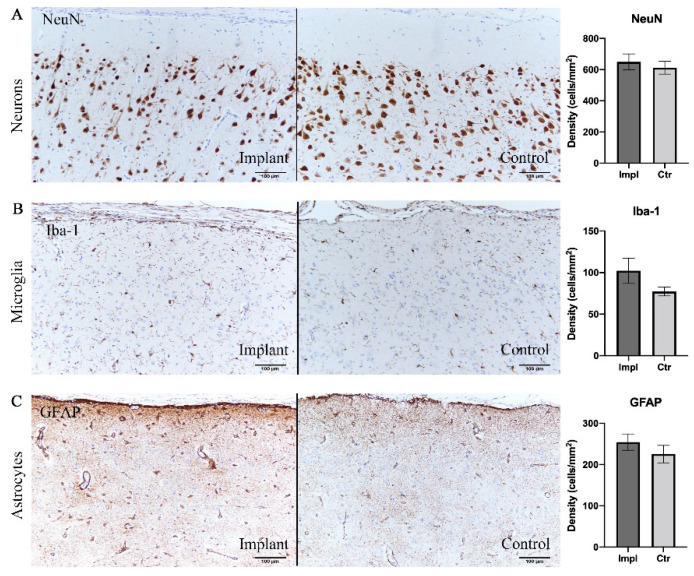
Immunohistochemical analysis demonstrating no significant difference in cell densities between implant sites and control sites. (**A**) Neurons labelled with NeuN antibody. (**B**) Microglia labelled with Iba-1. (**C**) Astrocytes labelled with GFAP. Scale bar = 100 µm.

To evaluate the intracortical reaction under the grid electrodes, immunohistochemical analyses were conducted on both implanted and control sides. Pathological observation of contralateral hemisphere brain tissue did not show inflammatory reaction or abnormal cell morphology. Assessments of the density of neurons, astrocytes, and microglia are shown in Table 1 and Figure 5. There was no significant difference between the implanted and control sides in the density of neurons stained with the NeuN antibody (*p* = 0.41, *t*-test), reactive astrocytes labeled by GFAP antibody (*p* = 0.40, *t*-test), and microglia labeled by Iba-1 antibody (*p* = 0.12, *t*-test) (Figure 5). Furthermore, reactive gliosis and reactive astrocytosis, which both reflect the endogenous responses to cortical tissue damage, were not observed; on both sides, the microglia maintained qualitatively similar morphology. In summary, these results demonstrated that little to no cytological change occurred in the cortex beneath the subdural ECoG electrodes. 

## 4. Discussion

### 4.1. Biocompatibility

We examined the chronic inflammatory tissue response 6 months after implantation of a fully implantable wireless device including subdural grid electrodes. Macroscopic observation and histological investigation revealed typical inflammatory reactions around the titanium casing and silicone-covered charging coil unit and epidural connective tissue formation over the dural membrane. The fibrosis, inflammatory cell infiltration, and angiogenesis in the tissues surrounding subcutaneously located devices are thought to be normal responses reflective of postoperative tissue repair and chronic reactions to implants [22]. The devices were stably implanted for 6 months with no severe infections or other complications. In this study, we did not evaluate the response longer than 6 months. Taking our clinical experiences in device implantation into consideration, generally, there are no major changes later than 6 months with respect to biocompatibility and safety. So we think 6 months’ implantation is sufficiently long to evaluate biocompatibility and safety. However, from a cosmetical standpoint, dissected muscle atrophy continues frequently even later than 6 months and sometimes up to several years. This is apparent from appearance changes but does not affect the recording performance.

Increased thickness of the dural membrane and fibrous encapsulation of the electrode array that were found in this study have been widely reported in other chronic ECoG implantation studies for both epidural and subdural placement [23,24,25,26]. We further quantitatively measured the thickness of the reactive tissues in the subdural space and found statistically significant differences compared with the control dural membrane. Results showed the dorsal encapsulation was thicker than both the ventral encapsulation and the control dural membrane. Consistent with previous studies in epidurally implanted ECoG electrodes [24,25], we demonstrated that the fibrosis encapsulation tissue proximal to the dural membrane was significantly thicker than that distal to the dural membrane for subdural electrode arrays. The subdural connective encapsulation tissue reported here would be expected of a long-term foreign body response to implants and/or a result of the acute wound healing reaction [16,27]. During the implantation surgery, the procedures of resection of the dural membrane, insertion of the ECoG array, and suturing of the dura cause tissue damage, thus leading to pro-inflammatory factor expression and anti-inflammatory/pro-wound healing reactions [28]. Several experiments demonstrated an increase in dural membrane thickness following craniotomy and dural incision [27,29,30]. Dural incision inevitably causes dural tissue damage and microbleeds and may lead to greater tissue damage than that caused by array insertion or dural suturing. Minimizing the dural incision might lead to minimization of the thick dorsal encapsulation. By contrast, different from the dorsal encapsulation, the ventral parts showed minimum encapsulation with no statistically significant difference from the control dural membrane. This would be expected as the result of minimum chronic foreign body response to the implants. A previous study reported that the dorsal portion of the encapsulation emerged from the original dural membrane, while the ventral portion grew de novo following implantation [24]. This would explain the different thickness of connective tissues and different mechanisms of fibrogenesis.

Several methods were tested to mitigate this inflammatory response by changing the material or mechanical properties of the electrode array or coating it with biological agents [14]. However, studies showed that dural thickening may have been an inevitable consequence of craniotomy and durotomy that was exacerbated by the presence of a foreign body [27,29]. Despite the encapsulation, the ECoG electrodes were extracted from the subdural space with little effort, indicating relatively few adhesions to the cortical surface. This could be attributable to the high degree of smoothness and non-porous surface features of the silicone sheets that covered the electrodes, which are less susceptible to fibrosis and adhesion formation [31]. 

For subdural electrode implants, only the leptomeninges separated the brain from the electrodes. Microscopic observation of the arachnoid membrane under the implanted array showed a downward compressive deformation caused by the occupying effect of the electrodes. However, HE staining also demonstrated the continuous integrity and normal structure of the leptomeninges, indicating that the BBB was not disrupted by subdural implantation. Studies have shown that chronic BBB breach causes local inflammation and is a critical determinant of electrode recording function [32]. The intact structure and function of the BBB are important for protecting brain tissue and prolonging the recording stability of ECoG devices. We found some slight yellow pigmentation and minimal inflammation on the array surface, which demonstrated microbleeds and pro-fibrogenic factors in the subdural microenvironment. However, these minor factors from the subdural or epidural space could be blocked from flowing into the cortical tissue because of the existence of the leptomeninges layer. 

The effect of subdurally implanted electrodes on neurons, astrocytes, and microglia was investigated by comparing to those in the contralateral hemisphere. Microglia and astrocytes are the two main glial cell types in the brain tissue and are generally kept in resting or “ramified” states. In response to tissue stimulation or injury, these cells become reactive and undergo proliferation, thus inducing an inflammatory reaction to protect the cortex [33,34]. In order to investigate the proliferation of these cells, we counted the densities of cells stained with NeuN, GFAP, and Iba-1 antibodies in the cerebral cortex bilaterally. Our results showed no statistically significant difference in cell densities between sides. In addition to the proliferation, activated microglia undergo a morphological change from a branched into a more compact state, while activated astrocytes become hypertrophic [33,34]. In this study, the cytomorphological characteristics of the cerebral cortex under the electrodes were not affected and were consistent with those in the contralateral hemisphere. Our neuronal density results in both hemispheres agreed with those of previous studies on rats, macaques, and human beings, which showed a mild inflammatory process with no significance in the brain region covered by subdural/epidural electrode arrays after 30 to 666 days [22,24,25,35]. Thus, considering the depression caused by the compression from the newformed dura fibrosis, it is plausible that the slight increase in cell density is most probably due to the pervasive mechanical deformation of brain tissue [36] rather than a response to foreign body inflammation. 

Compared with epidurally implanted electrodes, subdural implantation results showed similar inflammatory responses and biocompatibility. We believe that complete separation from the epidural space using water-tight dural closure reduces inflammatory factors in the subdural space where the electrodes are placed, thus mitigating the foreign responses to the device and increasing its long-term performance. However, in this study, we only measured the quantitative thickness of the subdural reactive tissue and were not able to collect data regarding epidural tissue proliferation. A limitation of this study is that we could not compare the degree of the inflammatory reaction between the epidural and subdural space. Another limitation is that the leads were implanted between the electrode array and the dural membrane, which could be a factor increasing the foreign body response and the dorsal encapsulation thickness. Thus, not placing the leads over the subdural electrodes might be a better choice to alleviate the subdural inflammatory reaction. Further experiments are needed to confirm this speculation. Moreover, it is methodologically optimal to set a sham control group. Instead we used a self-control method that compares inflammatory responses such as immunohistochemical factors and fibrosis thickness between the implanted hemisphere and the contralateral hemisphere without implantation. The finding that immunohistochemical analyses showed no inflammatory reactions in the contralateral hemisphere means that we did not need a sham group as a result. This self-control method can eliminate the effects of individual difference that are expected as larger than right and left differences in a subject. This may also contribute to reducing the number of animals, which is another important factor of animal experiments. Finally, there is a limitation of the current half-sized model of the real device. It is not sure whether the half-sized model for beagle dogs is exactly the same as the real device for humans, although it is at least better than the real model for beagle dogs that is too large to evaluate safety and biocompatibility of the device.

### 4.2. Half-Sized Device and Beagle Model

In the present study, the half-sized device was implanted in the dog’s skull. In clinical application, the real-size device is intended to be placed into the space of the removed bone flap made by craniotomy. So, we compared the implantable devices placed into the removed bone space (Table 2). There is only one skull-implantable device that is already medically approved: RNS. There are a few skull-implantable devices for BMIs previously reported [25,37,38]. The sizes of these devices are similar and small enough to be implanted in the large human skull, but too large to be implanted in the most of the experimental animals’ skulls. Here, we used the half-sized model of the implantable device to evaluate safety and biocompatibility.

Several animal models have been established in preclinical research because of their relatively suitable skull sizes for implanted devices [23,24,26,39,40,41,42]. Minipigs and sheep have been used in studies of cuff electrodes and the WIMAGINE epidural ECoG system [25,43] to mimic the implantation of clinical devices. However, because the frontal sinus of a minipig is a widely open cavity that communicates with the paranasal sinuses and the thickness of the sheep skull and curvature of the sheep brain are quite different from those in humans, it is difficult for neurosurgeons to perform the craniotomy and implantation [25]. Moreover, considering the cost and ethics of studies using macaques, their use should be limited to evaluations of efficacy that require intelligent tasks. Therefore, we chose the beagle dog breed as the experiment model for long-term implantation, further evaluation of long-term stability of signal quality, and evaluation of efficacy that requires simple movement tasks. The designed titanium case (18 × 27 × 8 mm) and silicone-covered coil unit (20 × 25 × 5 mm) were fixed on the frontoparietal bone, and craniotomy was performed on the left side, thus not crossing the midline, avoiding additional manipulations. We did not encounter much difficulty during surgical procedures in the subdural implantation of electrode arrays over the somatosensory cortex. Postoperative observation of motor behavior showed normal coordination and movements of limbs for all dogs, indicating normal sensorimotor cortex function after the implantation of electrodes [44]. Moreover, these animals are easy to handle and are submissive to humans, which makes it feasible to perform further safety and efficacy evaluations in clinical trials of implantable BMIs.

Although the beagle model took advantage of the adequacy of skull size and correspondence between the electrode array planner surface and brain curvature, there were some limitations. One is that it is difficult to water-tightly suture the thin dural membrane of beagles, even when using a microscope, although it is easy to perform in the thick human dural membrane without any use of microscopes. Future studies should improve the surgical procedures in order to ensure water-tight closure. Another limitation is that the motor area in beagles is located under the prefrontal bone near the orbit [45]. This anatomical feature makes it difficult to sufficiently cover the motor cortex with standard quadrate-shaped grid electrodes. Further development of suitable electrodes that can be slipped into the tip of the dog’s small frontal lobe is required.

This study is one of the steps taken toward the preclinical validation of long-term ECoG recording device implantation. Minimal inflammatory responses found in the beagle model suggest sufficient biocompatibility and chronic stability of subdural electrodes. Moreover, the fully implantable wireless device presented here allowed BMI application in daily activities. In addition to its use as a recording modality for BMIs, ECoG has increasingly become a neuromonitoring method in a variety of neuroscientific fields, particularly in awake, freely moving animals. Here, we established a beagle model for wireless subdural ECoG recording for the histological evaluation of chronically implanted devices, further nonclinical evaluation of safety and efficacy, and varied neuroscientific research under unconstrained conditions.

## 5. Conclusions

We implanted a half-sized model of a fully implantable wireless device for subdural ECoG recording in beagles. Histological evaluation after 6 months’ chronic implantation showed a typical and expected chronic tissue reaction in the subcutaneous and epidural spaces, minimal chronic tissue reaction in the subdural space, and no chronic tissue reaction in the cortical tissue. Our results following long-term implantation confirmed the reliability and compatibility of the approach and could be a first step to further development and technological transfer toward clinical BMI applications. Additionally, we established a beagle model for subdural ECoG recording study. This methodology could be used in not only preclinical evaluation but also neuroscientific research, particularly for experiments examining freely moving, awake animals.

## Figures and Tables

**Table 1:** Densities of positive cells.

	Site	Cells/mm^2^	*p*
NeuN	Impl	649.5 ± 125.5	0.41
	Ctl	612.0 ± 100.8	
Iba 1	Impl	102.2 ± 52.0	0.12
	Ctl	77.2 ± 13.0	
GFAP	Impl	254.5 ± 48.8	0.40
	Ctl	225.5 ± 52.0	

Impl: implanted cortex. Ctl: control cortex. NeuN: neuronal nuclei staining. Iba 1: ionized calcium-binding adapt molecule 1. GFAP: glial fibrillary acidic protein.

**Table 2:** Comparison of devices implanted in the skull.

Device	RNS	Brown University MEA ^1^	WIMAGINE	Real Size Model of This Study
use	epilepsy treatment	BMI	BMI	BMI
electrode location	Intra-cortical/subdural	Intra-cortical	Epidural	Subdural
Recording channel	4	100	64	64
Stimulation channel	4	-	-	-
Size	60 × 27.5 × 7.5 mm	50 × 40 × 10 mm	50 × 50 × 10 mm	50 × 30 × 10 mm

^1^ MEA: microelectrode array.

## Data Availability

Data sharing not applicable.
